# Utero-Cutaneous Fistula in Ruminants: Characterization of the First Cases in Ewes and Cows

**DOI:** 10.3390/ani14020344

**Published:** 2024-01-22

**Authors:** Antonio Carlos Lopes Câmara, Gustavo Peixoto Braga, Andrei Antonioni Guedes Fidelis, Fausto Romualdo de Faria Dantas, José Renato Junqueira Borges, Márcio Botelho de Castro

**Affiliations:** 1 Large Animal Veterinary Teaching Hospital, College of Agronomy and Veterinary Medicine, Universidade de Brasília, Brasília 70910-970, DF, Brazil; jrborges@unb.br; 2 Independent Researcher, Brasília 70760-517, DF, Brazil; gustavobragamv@gmail.com (G.P.B.); faustoromualdo@hotmail.com (F.R.d.F.D.); 3 Animal Reproduction Department, Univerity Center of Brasília (CEUB), Brasília 70790-075, DF, Brazil; andreifidelis@gmail.com; 4 Veterinary Pathology Laboratory, College of Agronomy and Veterinary Medicine, Universidade de Brasília, Brasília 70910-970, DF, Brazil; mbcastro@unb.br

**Keywords:** fetal retention, Nelore cows, perforated abdominal wounds, sheep, uterine adhesions

## Abstract

**Simple Summary:**

This report characterizes, for the first time, utero-cutaneous fistula (UCF) in animals, affecting ewes and cows, thus contributing to the knowledge about this rare condition in pregnant ruminants. Two ruminants presented non-specific clinical signs on the right ventral abdomen before UCF formation, such as moderate depression, low feed intake, and weight loss. The lack of reports on UCF in animals suggests underreporting or absence of diagnosis. Therefore, further studies are required to ensure improvements in the diagnosis and knowledge of pathogenesis, aiming at prevention.

**Abstract:**

Reports on UCF in animals are still lacking in veterinary literature. Detailed clinical signs, laboratory findings, and follow-up information from the first cases of UCF in two ewes and two cows are provided. The cases occurred over a 12-year period. All ruminants presented a fistulous tract or perforated wound on the right ventral abdomen, emitting a foul-smelling secretion possibly associated with macerated fetal parts or placental remains. Laboratory findings included anemia, leukocytosis by neutrophilia, and hyperfibrinogenemia in one ewe, and hyperfibrinogenemia in one cow. Ovariohysterectomy and fistulectomy were performed in one ewe, while the other three ruminants were submitted for the removal of fetal parts and placental remains through the UCF. Two ewes died within 12–48 h, and the two Nelore cows had an uneventful recovery, achieving secondary intention healing within 30 to 35 days. As a never-reported or unnoticed disease of the reproductive tract, UCF is an unusual consequence of dead fetus retention in an end-stage pregnancy and a potentially life-threatening condition in ruminants, especially ewes. Further broad studies in large herds of cattle and small ruminant flocks must be conducted to estimate the incidence of UCF and ensure improvements in the diagnosis and knowledge of pathogenesis, aiming at prevention.

## 1. Introduction

In women, the vast majority of uterine fistulae result from abnormal communication between the uterus and the urinary bladder (utero-vesical fistula), vagina (utero-vaginal fistula), or intestines (utero-enteric fistula) [1,2,3]. Utero-cutaneous fistula (UCF) is an extremely rare variant of uterine fistulae that results from pathological communication amidst the anterior wall of the uterus and the abdominal wall [2,4]. The few UCF cases reported in women in the past 30 years were almost exclusively observed following surgical intervention, such as cesarean section or treatment for miscarriage [1,2,3,4,5,6,7].

Reports on UCFs in animals are still lacking in veterinary literature. Despite UCF being mainly reported in the post-partum period in women [1,2,3,4,5,6,7], we present four cases of UCF affecting pregnant cows and ewes from different locations in Brazil. We report on the first cases of UCF in any animal species and provide detailed clinical signs, laboratory findings, and follow-up information, thus contributing to the knowledge about this rare condition in pregnant ruminants.

## 2. Case Series Presentation

Case 1. An 8-year-old and 20 kg crossbred ewe from Mossoró County, Rio Grande do Norte State, Northeastern Brazil, presented depression and low feed intake in the past five days. The flock was released on a paddock to graze in its native pasture, and at night, the animals were housed indoors. Flock management included free access to mineral supplementation and semi-annual deworming. The owner noted the increased volume of the ventral abdomen, which was punctured, revealing approximately 40 mL of green-yellowish content with a fetid odor. The increased abdominal volume recurred, achieving higher dimensions, and the ewe was referred for hospital care after six days of clinical evolution (13 April 2011).

**Figure 1:**
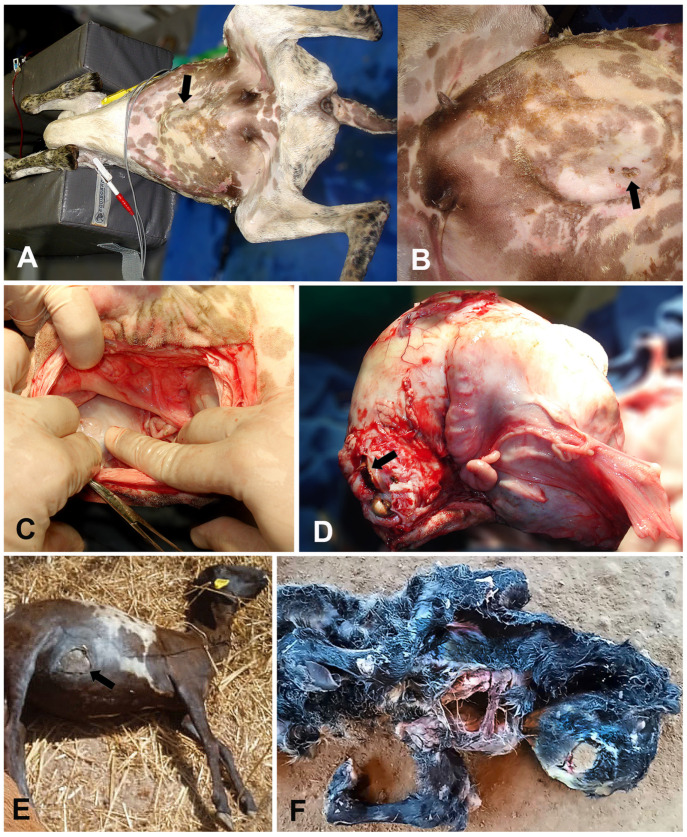
Utero-cutaneous fistula (UCF) in ewes. An 8-year-old crossbred ewe (Case 1. **A**–**D**). (**A**,**B**) (close view). Subcutaneous ventral-abdominal enlargement and a small draining tract with a brownish secretion (black arrows). (**C**). Celiotomy revealing the gravid uterus (surgeon’s right finger) and two fistulous tracts adhered to the abdominal wall. (**D**). Exteriorization of the uterus showing the fetal parts through the dissected location of the UCF (black arrow). A 3-year-old crossbred ewe (Case 2. **E**,**F**). (**E**) An 11 cm round skin wound at the right lateral ventral abdominal region (black arrow). (**F**) Arthrogryposis and a complete sternal cleft in a full-term malformed sheep fetus eliminated through the UCF.

Clinical examination revealed moderate depression, pale pink mucous membranes, dehydration (estimated at 8–10% by skin turgor), arched back, dull hair, tachycardia (164 beats per minute [bpm]), moderately empty, and hypomotile rumen. Rectal temperature was 38.9 °C (102 °F). During deep abdominal palpation, a firm structure was identified in the anatomical location of the uterus, and a brownish, foul-smelling secretion drained from the abdominal fistula, which was located 15 cm cranial to the udder, close to the midline of the abdomen (Figure 1A,B). Abdominal ultrasonographic evaluation (3.5-MHz convex transducer) detected the presence of a single fetus without heartbeats. Thus, the presumptive diagnosis was a UCF associated with fetal death, and surgical intervention was advisable. Hematology revealed anemia (hematocrit: 21%, reference values: 24–50%; red cells: 6.3 × 10^6^/µL, reference values: 8–16 × 10^6^/µL; hemoglobin: 6.7 g/dL, reference values: 8–16 g/dL), leukocytosis (23.6 × 10^3^/μL; reference range: 4–12 × 10^3^ leukocytes/μL) by neutrophilia (19.1 × 10^3^/μL; reference range: 0.4–6.0 × 10^3^ neutrophils/μL), and hyperfibrinogenemia (9 g/L; reference range: 1–7 g/L) [8]. Serum total protein was within reference values for sheep [9].

The ewe underwent surgery on dorsal recumbency for ovariohysterectomy and fistulectomy (Figure 1C). After pre-medication with 2% xylazine hydrochloride (0.15 mg/kg, intramuscularly [IM]) and morphine (0.1 mg/kg, IM), induction was obtained with propofol (5 mg/kg, intravenously [IV]). Orotracheal intubation was performed, and general anesthesia was maintained using 1.3 minimum alveolar concentration (CAM) of isoflurane and 50 mL/kg of 100% oxygen flow in a semi-closed circuit. After routine surgical preparation, a celiotomy was performed 5 cm cranial to the UCF. Hemostasis was accomplished by size 0 polyglactin ligatures on the ovarian pedicles, the uterine ligaments were transected, and the pregnant uterus was exteriorized from the abdominal cavity. The adhesions of the gravid uterine horn to the ventral abdominal wall were gently dissected, exposing fetal parts (Figure 1D). The abdominal cavity was then closed, and abdominoplasty was performed using size 2 polyglactin in a mattress pattern. Subcutaneous tissue was approximated with size 2-0 polyglactin, and dermorrhaphy was performed with size 0 nylon sutures in a Wolf pattern. Afterward, the UCF was dissected, presenting 7 cm in length and approximately 6 mm in diameter. The uterus was opened, revealing a macerated full-term male fetus.

Postoperatively, a broad-spectrum antibiotic (enrofloxacin: 5 mg/kg, IM, once a day [s.i.d.], for seven days) was applied, and anti-inflammatory (flunixin meglumine: 2.2 mg/kg, IV, s.i.d., four days) and analgesic (dipyrone: 20 mg/kg, IM, twice daily, four days) therapies were performed. The ewe died 12 h after surgery and was submitted for a necropsy. Pathological findings included pale mucous membranes, severe pulmonary congestion, and edema with scattered intra-alveolar fibrin aggregates; congestion and petechiation of most internal organs; and fibrin microthrombi in lungs and renal glomerular capillaries, thus supporting the diagnosis of shock, possibly as a result of sepsis.

Case 2. A 3-year-old and 35 kg crossbred ewe was purchased from another flock, and no previous reproductive data were available. At a new property in Santo Antonio do Descoberto County, Goiás State, Midwestern Brazil, the ewe was raised semi-extensively with access to native pasture and supplemented with corn silage. A veterinary practitioner diagnosed a right ventral abdominal eventration during the flock assessment for deworming and pregnancy diagnosis. The ewe did not present signs of pain or discomfort, and abdominal ultrasonography (3.5-MHz convex transducer) revealed a gravid uterus within the eventration content (hysterocele), that could be manually reduced. No further treatment was performed, according to the practitioner.

Forty-two days later (6 September 2022), the ewe was found with an 11 cm open wound purging a brownish fetid secretion with placental remains at the right lateral ventral abdominal region (Figure 1E). A full-term malformed male dead fetus showing arthrogryposis and a complete sternal cleft was found near the ewe on the ground of the stockyard (Figure 1F). The results of a vaginal inspection were unremarkable. Placental remains removal and uterine lavage were conducted through the UCF. Therapy with antibiotic (enrofloxacin: 5 mg/kg, IM, s.i.d., seven days) and anti-inflammatory (flunixin meglumine: 2.2 mg/kg, IV, s.i.d., four days) were instituted, but the ewe died 48 h later, and no necropsy was performed.

Case 3. A 5-year-old Nelore cow at a farm in Cachoeira de Goiás County, Goiás State, Midwestern Brazil, was raised on an extensive production system, feeding on *Urochloa decumbens* pastures and receiving mineral supplementation. The cow was inseminated in November 2021, and the expected calving period was September 2022. In mid-July 2022, the cow presented moderate depression, low feed intake, and progressive weight loss, and was treated with antibiotics (penicillin and streptomycin mixture) and vitamin B complex (oral route, s.i.d., continuously) by the owner. The cow recovered its appetite and commenced to gain weight. On the expected delivery date, there was no calving, and the event was considered a pregnancy failure or a miscarriage. Thus, the cow was discarded from the herd and taken to a fattening lot to be slaughtered after proper weight gain. There were no bulls or steers in this lot. 

**Figure 2:**
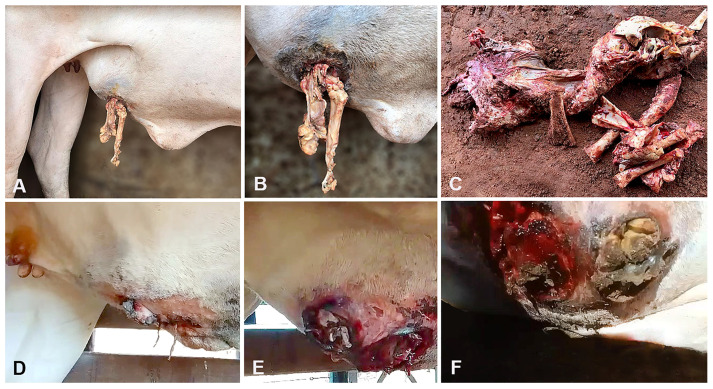
Utero-cutaneous fistula (UCF) in cows. A 5-year-old Nelore cow (Case 3. **A**–**C**). (**A**,**B**) (close view). UCF filled by fetal macerated soft tissues at the right ventral abdomen. (**C**). Bones and fetal macerated soft tissue remains after removal through the skin’s fistulous opening. A 7-year-old Nelore cow (Case 4. **D**–**F**). (**D**). An increased volume with a skin wound at the right lateral ventral abdominal region. (**E**,**F**) (close view). Fistulous tracts communicating with the uterus and filled with bones, fetal remains, and purulent content.

Eight months later (13 March 2023), the ranch employee observed a perforated wound on the right ventral abdomen with bone exposition during a routine check, and a veterinary practitioner was required for attendance. The cow showed a moderate body condition score (2.5/5), and all clinical parameters (rectal temperature: 38 °C [100.4 °F], ruminal motility, heart rate: 70 bpm, and respiratory rate: 24 breaths per minute) were within reference values. A 7 cm round fistulous tract was present on the skin at the right ventral abdomen, located 30 cm cranial to the udder and 15 cm lateral from the midline (Figure 2A,B). Fetal bones and macerated soft tissue parts were hanging out of the skin fistulous opening (Figure 2C) with a fetid odor, and the diagnosis of UCF was settled. Adherence between the uterus and the abdominal wall was detected upon rectal palpation, and the fistulous tract was estimated at 75 mm in diameter. After mild tranquilization (2% xylazine hydrochloride: 0.1 mg/kg, IV), the cow was restrained in left lateral recumbence, and remnants of a macerated fetus, soft tissues, and a foul-smelling uterine secretion were removed through the UCF. Therapy with antibiotics (long-acting 20% oxytetracycline: 20 mg/kg, IM, every 48 h, three doses) and daily topical wound treatment were performed. Daily cleaning consisted of washing the perforated wound with tap water and 2% chlorhexidine followed by topical antibiotic spray (Bactrovet^®^, Konig, Buenos Aires, Argentina), achieving secondary intention healing in 35 days. The cow was slaughtered 120 days later with a good body condition score (4/5), at 540 kg.

Case 4. During a routine pregnancy evaluation in the breeding season, a 7-year-old and 353 kg Nelore cow from Santa Rita de Cássia County, Bahia State, Northeastern Brazil, showed a severely enlarged uterus upon rectal palpation. The cow was raised on an extensive production system, feeding on *U. decumbens* and *Cynodon* spp. pastures, and was also given mineral supplementation. No fetus was detected during palpation, and rectal ultrasonography (5-MHz linear transducer) revealed a large volume of anechoic fluid accumulation within the uterus. The owner authorized no treatment. Fifty days later (7 July 2023), the cow showed an increased volume at the right ventral abdomen with a skin wound (Figure 2D). On a closer examination, fistulous tracts filled with fetal bones and purulent content were evident (Figure 2E,F). The Nelore cow received a similar treatment with antibiotics and daily topical wound treatment as in Case 3.

Blood samples were collected four days after the procedure, and laboratory changes were restricted to hyperfibrinogenemia (8 g/L; reference range: 1–7 g/L) [8] and hypoalbuminemia (1.7 g/dL; reference range: 3.03–3.55 g/dL) [9]. Leukogram, erythrogram, urea, creatinine, globulin levels, aspartate aminotransferase, creatine phosphokinase, and gamma-glutamyl transferase activities were unremarkable [8,9]. The wound from the UCF achieved secondary intention healing in 30 days. The cow was slaughtered 150 days later with a good body condition score (4/5), at 450 kg.

## 3. Discussion

UCF is a very rare complication of cesarean section in women, with about 20 cases reported in the literature in the past 30 years [1,2,3,4,5,6,7,10,11,12]. The pathophysiology of UCF is not well understood [2], but most UCFs develop secondary to post-partum or postoperative complications, such as cesarean section or treatment for miscarriage in women [1,2,3,4,5,8,9,10,11]. Additionally, a few reports of UCF are secondarily associated with endometriosis [10], intramural fibroids [12], and leiomyoma [5]. Other risk factors associated with UCF formation include septic abortion, uterovaginal malformations, multiple abdominal surgeries, pelvic actinomycosis due to intra-uterine devices, prolonged use of drains, incomplete closure of incisions, pelvic abscesses, incomplete placenta removal, and curettage [1,2,3,4,5,8,9,10,11,12].

To the best of our knowledge, we are reporting UCF in pregnant animals for the first time. In ruminants, the most common causes of abdominal hernias are infections in the navel area [13], severe blunt trauma, overstretching or tearing of the abdominal muscles, and congenital defects in the abdominal wall [14]. In the cases observed in sheep and cows herein, abdominal trauma was suspected to be the leading cause related to UCF, as observed in one sheep with a previous hysterocele (Case 2). 

The pathogenesis of UCF is not well determined, but predisposing factors include consecutive cesarean sections [2], incomplete closure of abdominal incisions, placement of abdominal drains, multiple abdominal surgeries, partial placenta removal, septic abortion, pelvic abscesses, pelvic actinomycosis due to intra-uterine devices, uterine curettage, dystocia and use of forceps in women [3,4,11]. In the cases observed in ruminants, we hypothesize that UCFs were a result of abdominal trauma, promoting damage to the uterine serosa and/or stretching of the pregnant uterus, enabling adhesions between the uterus and ventral abdominal wall. A previous study on small ruminants pointed out the possibility of blunt trauma causing muscle and visceral disruptions or contusions without making any external wound [14]. In our cases, UCF occurred on the right ventral abdomen (Cases 2–4) or near the midline (Case 1), probably due to the physical barrier promoted by the rumen. In addition, it has been proposed that uterine adhesions prevent uterine mobility within the abdominal cavity, resulting in decreased cervix dilatation and expulsive forces during labor and fetal retention [15,16,17]. Following fetal retention, fetal death and maceration are expected. A dead fetus within the uterus might promote local inflammation and adherence between adjacent tissues, with devitalization and necrosis of the uterine and abdominal wall tissues. Consequently, uterine injury and abdominal enlargement might lead to a focal disruption, with the formation of the fistulous tract between the adhered uterus, abdominal wall, and skin, which might explain the gross changes detected. Both the action of gravity on the dead fetus and devitalized uterine tissues, and the anatomical location of the rumen on the left, have probably influenced the ventral and right ventrolateral UCF location observed in all cases herein.

It is important to note that one sheep and one cow (Cases 1 and 3) with UCF showed non-specific clinical signs, such as moderate depression, low feed intake, and weight loss. In addition, a previous hysterocele was detected in one ewe (Case 2) with no apparent systemic disturbances, while no clinical changes were observed in the second cow until UCF arose (Case 4). These clinical features demonstrate the need for improvement in monitoring herds by owners and keepers, especially in recording reproductive data, such as mating date and expected birth.

In a previous report of a pregnant ewe with depression, a large mass palpated in the ventral abdomen was a dead intact fetus that was delivered via cesarean section 33 days after the co-twin birth [15]. Additionally, a tight adherence between the gravid uterine horn and the abdominal wall surrounded by a purulent inflammatory rim was detected [15]. These circumstances might evolve similarly toward a UCF formation or even death, as observed in two ewes that died after surgical procedure (Case 1) or fetal removal (Case 2) in our study, probably due to secondary bacterial infection [15,16]. In Case 1, hematology revealed severe neutrophilic leukocytosis without immature granulocytes and hyperfibrinogenemia, suggesting the release of these cells from the marginal to the circulating pool, a typical response of ruminants to severe inflammatory processes and bacterial infections [8]. 

Contradistinguished from both ewes with a fatal outcome, the Nelore cows had an uneventful recovery after fetal removal through the UCF, achieving secondary intention healing within 30 to 35 days. Hyperfibrinogenemia detected in Case 4, in addition to the unremarkable hemogram, might suggest a local inflammatory response to abdominal and uterine tissue injuries and adhesions [18]. Both cows remained in their herds without any clinical changes until they gained enough weight to be slaughtered. Contrasting with the difficult diagnosis in ruminants, who mainly demonstrated an increase of volume in the ventral abdominal region, affected women have shown bloody discharge through the abdominal skin in a Pfannenstiel scar (a scar associated with intra-abdominal adhesions) during menstruation, a pathognomonic clinical sign of the UCF [1,2,3,4,5,6,7,10,11,12]. 

Clinical findings have aided the diagnosis of UCF in women through imaging exams, such as magnetic resonance imaging, considered the gold-standard diagnostic test, computed tomography scans with intravenous contrast, hysterosalpingography, and fistulography [2,3,4,5,10]. A foul-smelling secretion draining from the abdominal fistula and ultrasonography in one ewe (Case 1), and fetal parts insinuated through the fistulous abdominal wound in the other cases, supported the definitive diagnosis of UCF in this study. 

This report provides knowledge on ruminants’ theriogenology and obstetrics and suggests UCF as a potentially life-threatening condition for pregnant animals. The main limitations of this study were a lack of standardization in the clinicopathological investigation and therapy, and reliance on veterinary practitioners’ information and photos in real field conditions. All findings reported herein add peremptory novelties on UCF to the veterinary literature.

## 4. Conclusions

As a never-reported or unnoticed disease of the reproductive tract, UCF is an unusual consequence of dead fetus retention in an end-stage pregnancy and a potentially life-threatening condition in ruminants, especially ewes. Further broad studies in large herds of cattle and small ruminant flocks must be conducted to estimate the incidence of UCF and ensure improvements in the diagnosis and knowledge of pathogenesis, aiming at prevention.

## Data Availability

The data presented in this study are available on request from the corresponding author.
